# “We Are Now Free to Speak”: Qualitative Evaluation of an Education and Empowerment Training for HIV Patients in Namibia

**DOI:** 10.1371/journal.pone.0153042

**Published:** 2016-04-07

**Authors:** Ellen W. MacLachlan, Katy Potter, Ndapewa Hamunime, Mark G. Shepard-Perry, James Uusiku, Ricky Simwanza, Laura J. Brandt, Gabrielle O’Malley

**Affiliations:** 1 PATH, Reproductive Health Program, Seattle, Washington, United States of America; 2 International Training and Education Center for Health (I-TECH), University of Washington Department of Global Health, Seattle, Washington, United States of America; 3 Namibia Ministry of Health and Social Services (MoHSS), Windhoek, Namibia; 4 International Training and Education Center for Health (I-TECH) Namibia, Windhoek, Namibia; University of Rochester, UNITED STATES

## Abstract

Although numerous studies provide evidence that active patient engagement with health care providers improves critical outcomes such as medication adherence, very few of these have been done in low resource settings. In Namibia, patient education and empowerment trainings were conducted in four antiretroviral (ART) clinics to increase patient engagement during patient-provider interactions. This qualitative study supplements findings from a randomized controlled trial, by analyzing data from 10 in-depth patient interviews and 94 training evaluation forms. A blended approach of deductive and inductive coding was used to understand training impact. Findings indicated the trainings increased patients’ self-efficacy through a combination of improved HIV-related knowledge, greater communication skills and enhanced ability to overcome complex psychosocial barriers, such as fear of speaking up to providers. This study suggests patient empowerment training may be a powerful method to engage HIV patients in their own care and treatment.

## Introduction

Like many countries in Southern Africa, Namibia has been severely impacted by the HIV epidemic. An estimated 220,000 adults are currently living with HIV and the prevalence is approximately 14.3% [1]. Over the course of the last decade however, Namibia has made significant progress scaling up antiretroviral therapy (ART), increasing access to life-saving treatment for people living with HIV (PLHIV). ART provision began in Namibia’s public sector facilities in 2003. As of 2013, Namibia had already achieved one of the highest ART coverage rates in sub-Saharan Africa with 87% of eligible adults and 70% of eligible children receiving ART, for a total of 113,486 people [2].

While ART scale-up efforts have enormous potential to impact the course of the epidemic, patient retention in care and adherence to treatment remain challenges. In 2013, an ART adherence baseline survey estimated patient adherence rates at all of Namibia’s public health facilities offering ART. The survey determined only 7.3% of patients were categorized as having high adherence to ART (95% adherence or above) [3]. A 2014 publication found Namibia’s retention in care rate of adults on ART after 12 months was 82%, with a range of 64% to 100%, depending on the site. Less than half of all adult ART sites met the target of ≥85% retention at 12 months [4]. Achieving high adherence is critical because it is associated with sustained HIV suppression and improved life expectancy, [5] reduced risk of developing resistance to antiretroviral (ARV) drugs [6] and decreased risk of transmitting HIV [7].

Recent literature from sub-Saharan Africa highlights the importance of patient-provider interactions in retention and ART adherence. Studies found that weak patient-provider relationships [8, 9] lack of information and understanding about ART (either information not provided or information misunderstood) and disrespectful or harsh treatment by providers, were all barriers associated with decreased retention and adherence [10, 11]. Namibia’s ART baseline survey cited above identified similar barriers to adherence, including challenges with patient-provider communication, lack of adequate communication materials, and patients not understanding ART-related instructions. The inverse has also been shown to be true: positive patient-provider interactions were linked to increased patient retention and adherence. For example, a 2014 study in Kenya found a higher number of patient-perceived physician communication behaviors were associated with an approximately 25% lower likelihood of patients missing an appointment and a 32% lower likelihood of patients missing medication [12]. Studies from Zambia, Tanzania and Ethiopia have shown that knowing providers personally, trusting them and receiving education about ART, motivated patients to take their medication as prescribed [11, 13, 14].

The evidence from studies outside of sub-Saharan Africa shows similar findings. Multiple studies in the United States have demonstrated a significant correlation between ART adherence and patients’ perceived level of engagement and satisfaction with their providers. In one study clients who were more engaged with their providers missed fewer appointments and reported higher adherence to their medication regimen and provider advice [15]. According to Flickinger, Saha, Moore, and Beach, [16] patients were more likely to keep appointments when they felt their providers “knew them as persons,” treated them with respect and dignity, explained things in ways they could understand and listened carefully to them.

I-TECH (International Training and Education Center for Health), at the University of Washington, has been working in Namibia since 2003 as a technical partner to the Centers for Disease Control and Prevention (CDC) and Ministry of Health and Social Services (MoHSS) to support the roll-out of ART. Through interactions with Namibian clinicians, leaders of HIV support groups and others, we learned that patients are often non-communicative during their clinical consultations, even when HIV clinicians encourage patient involvement. This can result in health care providers (HCPs) not providing necessary information to the patient, or overlooking side effects or other challenges the patient is experiencing. We hypothesized that addressing this lack of engagement through patient education and empowerment trainings for ART patients would help to improve patient-provider interactions.

The need to enhance patient-provider interactions in sub-Saharan Africa, and particularly Namibia, has received limited research attention. Most interventions approach the issue from the provider perspective, seeking only to improve the communication skills of the HCP. There are few studies that aim to empower the patient directly by building his capacity to convey and address his needs during consultations [17]. Against this backdrop, we developed a two-pronged research agenda: the first was to determine the effects of patient education and empowerment trainings on patient-provider interactions through a randomized controlled trial (RCT) in four ART clinics in Namibia; the second, presented in this article, was to enrich the RCT findings by examining individual patient perspectives on effectiveness of the trainings through a qualitative study.

### Intervention

The intervention consisted of a series of patient training sessions designed to increase patient engagement during clinical consultations. Three 2-hour training sessions on active participation, patient empowerment and communication were provided to groups of 4–8 adults newly initiating ART at four facilities in Windhoek, Rundu (in the far North), Onandjokwe and Katima Mulilo. The training curriculum was developed by I-TECH in Namibia with the local context in mind and content was translated into the languages spoken at each participating site. The same group of adults attended all three training sessions together and attended the ART clinic for follow up on the same days of the three training sessions.

The curriculum is informed by Bandura’s social cognitive theory (SCT) and particularly, the central construct of self-efficacy [18]. SCT assumes learning occurs in a social context through observation and modeling of others’ behaviors, and that adoption of modeled behaviors is determined by the observer’s degree of self-efficacy, or confidence in his/her ability to perform the modeled behavior. According to SCT, models should be well-regarded individuals with whom observers self-identify. To this end, the intervention was designed to train HIV-positive Namibians as facilitators. The theory also suggests that goal-setting directs behavior through increased self-efficacy and motivation, particularly when the outcomes are viewed as valuable. Thus the empowerment trainings incorporated activities to help patients set and reach their individual goals. Key to SCT is the notion that repeated practice of the learned behavior leads to skill acquisition [19]. Therefore, all three sessions were highly interactive, using a variety of hands-on methods for patients to practice the desired behaviors and gain competence and confidence. A summary of session contents is as follows:

Session 1: “Learning to Speak to Providers” begins to teach patients how to ask questions and explain their symptoms to doctors. A game is played to teach patients about HIV, ARV side effects and adherence issues. They discuss what it means to be an active patient and practice asking questions using the “Question Tool” (S1 Fig). They also set personal communication goals for future doctors’ consultations.

Session 2: “Using Tools to Help Communication” presents different tools to patients to help them prepare for consultations with their doctors, such as the “Body Map Tool” (S2 Fig) and the “Side Effects Checklist” (not shown). A “Pain Scale” (not shown) is also introduced to help patients think about how to communicate pain more effectively to their doctors. Patients are given scenarios to practice using all of the tools together to better communicate health concerns to a doctor.

Session 3: “Overcoming Barriers to Communication” helps patients think through any outstanding barriers to communication and strategize on how to resolve them. Patients practice assertive communication skills using role plays and the “I Tool” (S3 Fig), a tool to help patients address their specific health information needs. They finish with a discussion on mitigating language barriers with doctors.

In between each session, patients typically attended an ART clinic visit, which they debriefed at the beginning of Sessions 2 and 3. This design was intended to give patients a near-immediate opportunity for real life application of the knowledge and skills taught in the trainings, while also providing a systematic means to process patient experiences in a facilitated group setting. Facilitators were trained to offer specific feedback to patients on their improvements in order to enhance their self-efficacy and motivation.

### Randomized Controlled Trial

The effectiveness of the intervention was measured in a randomized controlled trial (RCT) of 589 patients. The intervention group received training sessions at approximately 2 weeks, 1–2 months, and 3–4 months after ART enrollment. Control group participants began their trainings six months after ART initiation. The Roter Interaction Analysis System (RIAS) for coding medical dialogue was used to code and quantify patient-provider conversations. RIAS outcomes were compared between study groups at 6 months. Statistically significant improvement was found in 5 of the 13 outcome variables for patient-provider interactions. Key findings included trained patients were nearly twice as likely to ask questions of their providers compared with untrained patients (adjusted difference in score .48, p = .012), and trained patients had greater positive patient affect and satisfaction with their providers than the controls (adjusted difference in score 2.08, p = .002). Doctors also gathered more information from trained patients (adjusted difference in score 2.96, p = .000) and showed a stronger positive emotional affect during encounters (adjusted difference in score .60, p = .02). A more comprehensive description of the RCT research design and findings were published in 2015 [20].

### Qualitative Evaluation of Trainings

As part of the RCT we conducted this qualitative study in order to enhance the interpretation of its findings by exploring individual patient perspectives on their training experience through in-depth interviews. Open-ended evaluation forms completed by facilitators on all three training sessions provided additional evidence on patient reactions to the training. Specifically, we aimed to identify how patients perceived the effects of the training intervention on their capacity to engage with providers and thereby understand more the underlying mechanism by which the trainings led participants to feel an increased sense of self-efficacy to be active and engaged patients.

## Methods

### Data Collection

#### Patient Interviews

Between February and June 2013, 10 in-depth interviews using semi-structured interview guides were conducted with trained patients at Onandjokwe ART Clinic (4 patients) and Katima Mulilo Hospital (6 patients) to understand if/how they were personally affected by the trainings. Patients were eligible to participate if they completed all three of the training sessions. Questions were open-ended and probing was used to investigate other topics of interest that emerged during the interviews.

Sampling was based on convenience and included any eligible patients who were already attending one of the two clinics for other purposes. Interviews were carried out on-site at the health facilities in a private setting. They were conducted in Silozi (Katima Mulilo) and Oshiwambo (Onandjokwe) by trained local facilitators (not the facilitators of the trainings), digitally recorded, transcribed and translated into English by the bilingual study site coordinators. Written informed consent was obtained from all participants to conduct and record the interviews.

#### Training Evaluation Forms

Hand-written evaluation forms were completed by the bilingual training facilitators immediately following the training sessions during the implementation of the RCT. The evaluation forms described facilitators’ perceptions of what worked well with the training and areas for improvement, and sometimes included summaries of reactions and quotes from individual training participants. During the RCT these evaluation forms were intended to provide facilitator feedback on the trainings in order to assure training quality. A total of 94 training evaluation forms (50 from Training 1, 32 from Training 2, 12 for Training 3) were collected during the course of the intervention, between January and November, 2012. Facilitators were asked to document the concepts participants appeared to appreciate most, areas they found unclear, topics they needed more information about and any participant concerns. Information gathered from the forms was used as “shadowed data” to supplement that of the in-depth patient interviews, as this permitted the inclusion of a wider breadth of perspectives on the trainings from beginning to end. Shadowed data also provide a better sense of the range of experiences beyond an individual’s personal experience [21]. Evaluation forms were completed in English and gathered from all of the study sites: Windhoek, Rundu, Onandjokwe and Katima Mulilo.

#### Data Analysis

The goal of analysis was to understand how the empowerment trainings affected patients’ capacity to engage with providers and their self-efficacy to be active patients. Scanned evaluation forms and transcribed interview transcripts were independently coded using Atlas.ti v.7.5.4 (Scientific Software Development GmbH, Berlin, Germany) and reviewed by a second researcher. Any coding disagreements were resolved through discussion. We used a combined technique of deductive and inductive thematic analysis. In the first coding phase, deductive codes were drawn from a review of the literature on patient self-efficacy and patient empowerment. In the second phase, inductive codes were established and applied to themes that emerged directly from the data [22].

### Ethical Considerations

This study received ethical clearance from the University of Washington Institutional Review Board (IRB) in March of 2011. It also received clearance from the Centers for Disease Control and Prevention IRB in July of 2011, and clearance from the Ministry of Health and Social Services in Namibia in January of 2011. The study commenced in January of 2012. Written informed consent was obtained from all individual participants included in the study.

## Results

### Study Population

Interviewed patients (N = 10) ranged in age from 28 to 42 years, with a median age of 30. Seven of the interviewees were female, 3 were male. The majority of patients received education through secondary school, while 2 had received some primary school education (Table 1). All of the patients had initiated ART during 2012 and had completed their third and final training session at least 1 month (range 1 to 8 months) prior to their interview. The completed evaluation forms represented over 200 trained patients across 94 different training sessions.

**Table 1:** Demographic Data of Interviewed Patients.

Patient #	ART clinic	Gender	Age	Education(highest level received)
1	Katima Mulilo	F	28	Secondary
2	Katima Mulilo	F	30	Primary
3	Katima Mulilo	F	31	Secondary
4	Katima Mulilo	M	30	Primary
5	Onandjokwe	F	30	Secondary
6	Onandjokwe	F	29	Secondary
7	Onandjokwe	F	40	Secondary
8	Onandjokwe	F	41	Secondary
9	Onandjokwe	M	30	Secondary
10	Onandjokwe	M	42	Secondary

### Overcoming Psychosocial Barriers

Patient interviews and evaluation forms revealed the difficult psychosocial barriers that patients had to overcome in order to actively participate in clinical consultations. Prior to the trainings, patients commonly described being afraid to “speak freely” and express their opinions during encounters. Some patients provided reasons why they feared speaking up, including a perceived lack of provider empathy for their condition, fear that doctors would get angry if they asked questions, and previous experiences in which doctors were “not open to talk to them when they ask questions” (P22, Session 1). As a result of the trainings, however, patients gained the courage to be more engaged and described feeling more “open” with providers.

*“According to me*, *these trainings helped me in communicating with the doctor because at first I was afraid to speak to the doctor but after these trainings it helped me how to express myself when I am with the doctor*, *they really prepared me*.*”—(P3*, *31 year-old female)*

*“I used to be afraid that maybe the doctor will get angry*, *but after the training it was easy*. *Even now I still have the courage of asking the doctor about my health*.*”—(P4*, *30 year-old male)*

*“Oh yes it [training] really helped me*, *the trainings gave me courage and good ideas that I never knew…I used to be very scared but not anymore*, *trainings have really done something good to me and now I am able to help other people like me who are also afraid to be free to talk to providers*.*”—(P5*, *30 year-old female)*

Other frequently described psychosocial barriers included feeling unsure of what to say to providers and lacking awareness of patient rights and responsibilities. Interviewed patients reported that the trainings provided them with the fundamental realization that they were ‘allowed’ to speak to doctors. Many were unaware they had the right to ask doctors questions related to their own health or be involved in health decision-making. Patients described how the trainings clarified patient-provider roles and helped alleviate their fear of speaking openly during medical encounters.

*“Talking from my personal experience I was always afraid to talk to providers at hospitals*, *even at this clinic*. *I did not know if I can tell the doctors about my feelings or the way I should be taking my medicine*, *but after this training I feel so free and open to talk to providers when I come for my follow-up*. *This training really helped me because I never used to be free to talk to nurses and doctors because I always thought that they are different from us*, *I never really used to be free and say all my complaints but after the training I know what to do*, *it helped me… Previously I just thought that the work of the doctors was just to make recommendations about my treatment without me asking them anything unless they ask me*.*”—(P5*, *30 year-old female)*

*“Previously I did not know if I could ask the doctor about my blood condition because I did not know if I can ask doctors questions*. *I now know that I can ask any questions anytime when I come to the hospital*.*”—(P8*, *41 year-old female)*

### Effects of Trainings on Patient Knowledge

All interviewed patients expressed that the trainings increased their knowledge of HIV-related topics, such as ARV side effects, strategies to improve adherence, and understanding their CD4 count. They explained how acquiring information on HIV and ART, often in conjunction with their acquired communication skills, promoted enhanced engagement with providers due to their ability to ask specific questions about their health condition.

*“I feel that the trainings have prepared me how to better communicate with the providers because now I can even ask the doctors and nurses about the level of the virus in my body and CD4 counts and they can tell me this information*.*”—(P9*, *30 year-old male)*

Evaluation forms suggested that participants had very low levels of knowledge regarding HIV prior to the training sessions. Facilitators described several participants who were previously unaware of why providers took their blood samples and what CD4 count meant. Other facilitators noted that participants felt frustrated because they had not been informed about ART’s negative side effects by adherence counsellors. Evaluation forms and interviews alike, revealed participants’ strong concern that most other PLHIV lacked the knowledge they had gained from the trainings. Data suggested participants placed a high value on this knowledge because they understood its importance to their health, including potential risks of lacking such information. As one facilitator reported, “They [participants] say many patients are not even aware of side effects so sometimes they stop to take their ARVs because of this” (P43, Session 1). Another facilitator from a Session 3 training wrote:

*“The participants said it’s good that they are part of this [training] they are just saying please do it for more patients so that we will be on the same page in our [understanding of] ARVs*. *We thank you for opening our hearts and minds*. *We are now free to speak*.*”*—*(P78*, *Session 3)*

Interviewed patients echoed similar sentiments:

*“I pity those who haven’t gone through these trainings because they know nothing*. *It was good that when one starts with the medications*, *he must go through these trainings so that they know what they should do*. *They are afraid to ask doctors on what they should do*.*”—(P4*, *30 year-old male)*

### Effects of Training Tool Use on Patient Skills

All interviewed patients felt that the trainings increased their ability to communicate with providers. They reported feeling more prepared to interact with doctors and to perform active patient behaviors, such as asking questions and reporting symptoms. Responses from interviewed patients about the tools were overwhelmingly positive, saying they appreciated them and found them easy to use. All patients also reported using at least one of the training tools (i.e., Body Map, Question Tool, Side Effects Checklist, Pain Scale, “I Tool”) during regular visits to their doctors since the trainings, and nearly all said they used two or more.

Patients described multiple ways in which the tools helped them communicate more effectively with their providers. For example, some patients found the tools useful because they helped to mitigate language barriers with their doctors. Multiple patients described successfully using the principles of the Question Tool to request an interpreter in order to better interact with the doctor. Another used the Body Map as a communication prop to circumvent language barriers.

*“I liked the body map most because it brings a communication link between me and the doctor especially when there is language understanding problem*.*”—(P5*, *30 year-old female)*

Several patients also explained how the Body Map, Pain Scale and Side Effects Checklist improved their ability to pay attention more to their pain, discomfort and other symptoms, as well as better communicate about these topics with their providers.

*“Pain scale activities have made us to understand our condition when we are sick*, *and the Body Map has made us to be aware and identify our body parts which need treatment*.*”—(P98*, *Session 3)*

*“It was easy [to use the Body Map and Pain Scale] because from there I found it easy to communicate well with my doctor*. *I could now tell him where I feel pain*.*”—(P1*, *28 year-old female)*

*“It was easy using this tool [Side Effects Checklist] because there was a time when I started medication that I was developing rashes on my body and I came to the hospital to see the doctor who stopped the medicine that I was taking immediately to allow recovery from the rashes and a new different medicine was given to me*. *I feel that the doctor responded well to the problem that I had*.*”—(P5*, *30 year-old female)*

The Question Tool was widely used by patients to elicit information from providers on topics like lab results, ARV regimens and adherence, and when to return for a follow-up visit. Patients said the tool helped them to think in advance about which questions they wanted to ask providers as well as to remember those questions during the visit.

*“It was very easy [to understand the questions] because they guided me in a straight way*. *These questions were important as it taught me to be prepared what I must ask the doctor*, *what I feel*.*”—(P4 30 year-old male)*

Some patients reported that the Question Tool enabled them to gather information to make more informed, health-related decisions, such as if/how it is possible to have a healthy child and how to maintain proper nutrition and hygiene.

### Patient Motivation

Most interviewed patients described their desire to be “treated well” by providers as motivation for adopting active patient behaviors. This was particularly evident in patient discussions on the use of training tools and their ability to facilitate enhanced interaction with providers. They suggested the tools were a means to receiving better quality care and treatment, and consequently, increased patient satisfaction with the medical encounter.

*“It was also easy to use the body map because if I’m sick or not feeling well on my body I have to show the doctor so that I get good treatment and so that I am happy when I get out of the consulting room*.*”—(P7*, *40 year-old female)*

*“[Using the Question Tool] I asked the nurse how much my CD4 counts were*. *I feel that the nurse answered me well…I have used the pain scale because I showed the doctor with my face that I was feeling well when I came for my clinic visit*. *If I was feeling sick I would still have showed him with my face that I was not well so that he or she treats me well*. *It was very easy to use the pain scale*.*”—(P10*, *42 year-old male)*

Even in situations where patients did not require the use of certain tools due to their health circumstances, they described being similarly motivated by the prospect of quality treatment.

*“I did not make use of all the tools that were in the trainings because there was not a day that I came to the clinic with a problem*. *However I do not have a problem to use them because I was trained and most especially if I want to be treated well*.*”—(P6*, *29 year-old female)*

### Active Patient Self-Efficacy

Active patient self-efficacy refers to a patient’s confidence in his/her ability to adopt active patient behaviors and engage with providers. Interview data suggest that all patients enhanced their active patient self-efficacy following the training sessions. Patients routinely stated they felt confident in their ability to engage with providers. They discussed overcoming their fears of speaking up to providers and feeling confident to speak freely and express opinions during consultations.

*“Yes*, *there are no challenges [that remain for me in terms of being an active patient]*. *I still urge those who will attend these trainings that they will not have a problem after the training…With me*, *I think there is nothing [else that I want to ask or say to the doctor but cannot]*. *When I will have a problem I will ask the doctor*, *that is the good part of these trainings*.*”—(P4*, *30 year-old male)*

“*They [patients] feel they are empowered and they are confident to ask anything to the health care workers*.*”—(P69*, *Session 2)*

*“The trainings prepared me very well to communicate with the nurses and doctors*. *I do not have any more problems…There is no other challenge [in terms of being an active patient] and all I need to do is to follow the doctor’s instructions and if I have any problem*, *then I should discuss with the doctor*.*”—(P10*, *42 year-old male)*

Some patients even demonstrated awareness that their efforts may be met with less than promising results from providers. Despite this, patients seemed confident that they would not be deterred from continuing to engage with providers in order to receive quality care and treatment.

*“I do not have any other challenges [in terms of being an active patient]*. *I’m so open to ask anything that I want to the providers especially those that have to do with my treatment*. *Even though sometimes the provider might not answer me very well I have to try by all means as an active patient to be well-treated*.*”—(P7*, *40 year-old female)*

### Adherence Self-Efficacy

Some interviewed patients described feeling more confident to adhere to their ARVs following the trainings, due to their enhanced understanding of how and when to take their medications. In some cases, patients specifically mentioned the usefulness of the adherence strategies they learned to remind them to take their pills. They also reported how their improved knowledge about ARVs, in addition to learning to be an active patient, motivated them to take their medications.

*“I think the whole training was useful because it gave us light and understanding how we should take our medications…It helped me on how I should take my medications and how to take them on time…In Training 1 I enjoyed much on how to take my medications*, *which means I enjoyed most because I now know how to take my medications and how to speak to my doctor*.*”—(P3*, *31 year-old female)*

*“What helped me or assisted me most was to be an active patient and to know how to take my medicine*. *It really motivated me as an HIV patient*. *It gave me strength to continue with my treatment and to know how to take my medicine accordingly…I remember where I was told how to remember taking my medicine like putting alarm on my cell phone or putting a pill drawn paper in front of my door so that I remember whenever I’m passing there especially when I wake up even though I did not here the beep of my alarm because the pill drawing in front of my bedroom door will alert me that I should take my tablets*. *That was the most important thing that I enjoyed from the trainings*.*”—(P7*, *40 year-old female)*

## Discussion

This qualitative analysis of patient education and empowerment trainings was conducted to supplement the RCT findings from the same intervention, whose results showed statistically significant improvements in the quality of patient-provider interactions for ART patients in Namibia. While the RCT determined the effects of the trainings on patients’ and providers’ communication behaviors, this study afforded a deeper awareness of the context in which behavior changes occurred. The joint implementation of qualitative studies with RCTs is useful and often necessary when quantitative methods alone are insufficient to evaluate complex behavioral interventions, particularly those with psychosocial components [23]. Qualitative studies allow for greater and more meaningful interpretation of trial findings, enabling researchers to unpack the intervention’s “black box” by understanding which aspects were effective and why [22].

Patient self-efficacy is widely recognized as a requisite factor for health behavior change, influencing not only the decision-making process but also the likelihood of initiating and sustaining behavior change [24]. The primary aim of this intervention was to increase the quality of patient-provider interactions via patient empowerment and education. Findings from this study strongly suggest the trainings enhanced patients’ self-efficacy to engage with providers during medical consultations. Increased self-efficacy does not always translate to actual behavior change; however, in this case, qualitative results are supported by RCT findings, which showed a statistically significant increase in trained patients asking questions of providers. Our findings resonate with those of a similar intervention in Indonesia, which found patient education and empowerment coaching led to improvements in active patient communication, due to increased patient confidence and communication skills to talk openly with providers [25]. Additional research is necessary to establish whether patient-targeted education and empowerment interventions can generate sustained benefits, especially for HIV/AIDS patients, who face a lifetime of care and treatment.

Our intervention’s theory of change (S4 Fig) proposed higher quality patient-provider interactions would ultimately lead to better ART adherence via enhanced patient adherence self-efficacy. This model is well-supported by the literature. A 2014 meta-analysis of 207 studies, reporting on over 100,000 ART patients, found self-efficacy to be the factor most strongly associated with adherence. Trust and satisfaction with the HIV care provider were also associated with greater adherence, especially in low and medium Human Development Index (HDI) countries [26]. Our qualitative and RCT study results serve to strengthen the evidence from these publications, as not only did trained patients have greater positive affect and express satisfaction with their providers’ responses, but doctors collected more information from patients and had a more positive emotional affect during consultations. This suggests empowered confident patients, motivated to be “treated well”, engaged more actively with providers. It is possible that this encouraged provider dialogue which, in turn, motivated doctors to collect more information from patients and, in the end, led to the provision of better care [27]. This is depicted in S4 Fig with the arrow from the physician pointing toward improving patient self-efficacy. Positive patient-provider interactions have been shown to generate greater adherence, due to patients’ feeling respected and understood [16] which subsequently enhances their confidence in their ability to maintain their treatment regimen [28].

Findings from this study suggest a major factor behind the intervention’s success was its three-pronged approach to improving patient engagement with providers, which included: 1) overcoming patients’ psychosocial barriers, such as the fear to “speak freely” with providers and “legitimizing patients’ right to speak,” [25] 2) improving patients’ HIV-related knowledge; and 3) enhancing patients’ communication skills. An important feature of the approach is the interdependent relationship between these training components, as each relies and builds upon the other in order to more comprehensively address patient training needs. For example, acquiring an understanding of one’s CD4 count (knowledge) is greatly enhanced by a patient’s ability to ask the doctor for his lab results (communication skills). Making such a request for results would not be conceivable for patients who are afraid to ask questions of their providers, or who do not understand that they have the right to do so (psychosocial barriers).

Our study findings support the hypothesis presented in the theory of change diagram (S4 Fig), which posits the three training components would collectively lead to an increase in patients’ self-efficacy to be active and engaged with providers during medical encounters. The need for a more comprehensive approach to address health behavior change is well documented. In order to enhance the likelihood of behavior change and achieving related health outcomes, there is evidence that it is necessary to go beyond traditional knowledge-based approaches and target patient understanding and self-efficacy [29]. Furthermore, in contexts where major psychosocial obstacles to patient communication exist, such as patient fear of questioning providers or lack of awareness of patient rights, our study contributes to existing research which proposes that addressing these barriers is essential [25]. By overcoming such obstacles, patients are given the opportunity to fully realize the potential of their acquired knowledge and communication skills. Similar to other studies, our findings suggest that clarifying patient-provider roles and explicitly “permitting” and encouraging patients to speak up to providers, were effective tactics [25, 30].

Another barrier that was discussed frequently by patients in this study was language. While patient-provider language barriers are not unique to Namibia, it is worth noting that a medical school did not exist in the country until 2010. As a result, the vast majority of its public sector ART doctors are non-Namibian and do not speak the native languages of their patients. Moreover, none of the 8–10 ART doctors who participated in our study was Namibian. This suggests there are likely cultural barriers in addition to the linguistic hurdles that patients face during consultations. A study conducted in South Africa found language and cultural barriers had adverse impacts on HCPs and patients, such as negative patient and staff attitudes towards each other, decreased quality of and patient satisfaction with care, and cross-cultural misunderstandings leading to poor patient-provider communication [31]. Further studies are needed to better understand how these factors affect patients’ experiences in Namibia.

A variety of training methods were used to enhance patients’ confidence to engage with providers. These methods align with Bandura’s suggested means of increasing self-efficacy in order to change behavior [18]. The trainings actively involved patients by giving them opportunities to practice active patient behaviors and process their experiences through constructive facilitator feedback, self-reflection and group discussion. This experiential approach for patient empowerment and education interventions is well-supported by the literature [32]. The trainings also included an exercise in Session 1 in which patients defined individual, communication-related goals for future consultations. At subsequent trainings, patients debriefed their consultation experiences with the group, including the successes or challenges in meeting those goals. The importance of goal setting as a method to empower patients and enhance self-efficacy is widely discussed in the literature, as it can help to gauge progress, strengthen patients’ sense of control, and motivate them to achieve the desired change [19, 32, 33]. HIV-related information in the trainings was taught using interactive techniques like the “knowledge game”, to keep patients engaged while providing them with important knowledge about their disease and treatment. A 2012 review of patient engagement interventions emphasized the need to use more participatory, patient-centered methods to increase health literacy and empower patients, versus the more traditional directive, advice-laden styles of teaching, which can lead to patient resistance or a sense of hopelessness [33]. The same review described how the provision of health information, via both written and oral means, can increase patients’ sense of empowerment, improve their ability to cope with challenges and may reduce anxiety [33]. Question prompts have also been linked to increased patient self-efficacy, reinforcing our findings from patient interviews, which suggest patients’ confidence was enhanced by using tools like the Question Tool. Finally, a sub-Saharan Africa-focused meta-ethnography found the use of patient groups as social support helped to strengthen HIV patients’ self-efficacy [34]. Our study assigned patients to training groups based on their ART initiation date and the same group makeup was maintained for all three training sessions. While information on the training groups was not specifically elicited from patients, many described appreciating the group discussions, “enjoying talking about our experiences in medication and HIV life” (P5) and numerous evaluation feedback forms remarked on patients’ high levels of engagement with the groups. Therefore, it is possible that these training groups functioned as patient support groups as well.

### Study Strengths and Limitations

Patients overwhelmingly reported a high level of satisfaction with the training activities. The few concerns mentioned by patients about the training sessions were regarding training length (that they should be longer than two hours and continue beyond three sessions) and translation issues. In order to convey information to patients in different parts of the country, who speak different languages, materials were originally developed in English and translated into local languages before the trainings by the training facilitators. Some patients remarked that their facilitators had a difficult time finding the correct words to use, which may have impacted the integrity of the materials’ content. Practically speaking, it also took more time to explain the content depending on the group’s translation needs, which likely resulted in less time for discussion or other activities compared to groups who required less translation.

The limited sample size and sampling methods of interviewed patients prevent generalization of the findings to other patients. Evaluation forms were not collected from every training and thus do not represent a complete universe of all trainings. Analysis of the evaluation forms and in-depth interviews did, however, reveal strong thematic saturation, suggesting the data’s rigor and reliability [35]. Use of project staff as facilitators to record data the training evaluations possibly introduced self-report bias, as facilitators may have been concerned that negative comments would reflect badly on staff performance. The high degree of agreement across all evaluation forms and the positive feedback from patients themselves makes this seem less plausible, particularly because facilitators tended to complete the forms collectively with patients, and often noted patient quotations directly (e.g., “We especially enjoyed the knowledge game”). We further attempted to minimize this bias by assigning the site coordinators, rather than the actual trainers, to facilitate evaluation discussions.

## Conclusion and Implications for Practice

This study, in combination with the RCT, showed that trained, Namibian ART patients increased their confidence and competence to actively engage with providers due to the patient education and empowerment intervention. These results may lay the foundation for new, innovative interventions for patients living with HIV in Namibia. For example, it may be practical and cost-effective to have existing community counsellors at clinical sites communicate with patients about increasing their participation in care and treatment. They might also be able to encourage patients to practice their right to speak to doctors, or lead sessions to teach patients how to communicate effectively with their health care providers.

The rapid scale-up of ART in Namibia has meant that more patients are seeking necessary care in an already overburdened health system. Lack of effective patient-provider communication can result in providers not offering some necessary information to the patient, or overlooking symptoms, side effects and other challenges that the patient is experiencing, but is hesitant to report. Given the incredibly limited time providers have with ART patients, empowering patients to use effective communication strategies may be the key to bridging this gap. Furthermore, this strategy can serve to prevent HIV-related complications as some may be avoided if adherence is supported and side effects addressed early through effective patient-provider interactions [36]. In this setting all patients had routine counseling and education sessions, both group and individual, for all patients on ART. Given our findings, this routine pre-ART counseling could be augmented to take into consideration patient empowerment support.

Patient empowerment training may also be a powerful way to engage patients in their own care and treatment. Without basic information about how to take their medication and manage their HIV disease, patients will not be able to make informed decisions about their lifestyle. In addition, feeling empowered to ask their providers specific questions about managing their disease is one way to improve patient engagement in chronic disease management. This training curriculum may be a promising resource for those who work with HIV/AIDS patients in resource-limited settings.

## Supporting Information

S1 Fig Question Tool. Questions depicted in the tool were for illustrative purposes only. During the ‘Patient Empowerment’ training sessions, patients were encouraged to develop their own relevant questions.
(DOCX)

S2 Fig Body Map Tool. (DOCX)

S3 Fig “I Tool”. (DOCX)

S4 Fig Theory of Change Diagram: Patient Education and Empowerment Training. (DOCX)
