# Supplementary material for: “We Are Now Free to Speak”: Qualitative Evaluation of an Education and Empowerment Training for HIV Patients in Namibia
Source: PLoS One. 2016 Apr 7;11(4):e0153042. doi: 10.1371/journal.pone.0153042 (PMC4824517; doi:10.1371/journal.pone.0153042)
Supplement: S1 Fig — Questions depicted in the tool were for illustrative purposes only. During the ‘Patient Empowerment’ training sessions, patients were encouraged to develop their own relevant questions. (DOCX) [file pone.0153042.s001.docx]

**Figure 1: Question Tool**

**Use these questions as a start to becoming an active patient. You can use some or all of the questions below during your visit with your doctor. Before asking any questions, first share with the doctor how you are feeling and any changes you have experienced since your last visit.**

General Health Status Questions

1. How do you think I am doing on my ART medication?

2. Do you see any changes that concern you?

Questions about Lab Work

3. What are my lab results (viral load, CD4 count)?

4. What do they mean?

5. How often will I take lab tests?

6. What tests and for what purpose?

7. When you take blood, am I tested for any other sexually transmitted infections?

Questions about Medication

8. What are the directions for taking my medication?

9. How do these medications work with my body?

10. What are the possible side effects?

11. What do I do if I get a side effect?

12. What if I feel worse once I start taking the medicine?

13. What do I do if I miss a dose?

14. What do I do if I run out of pills?

15. What will happen if I have a drink or take any drugs?

16. Can I eat or drink whatever I want while I am taking these medications?

17. When should I consider changing my treatment plan?

18. Are you prescribing me the same medications or changing any of my treatment regimen?

Additional Questions

19. What else can I do to stay healthy?

20. What happens if my child takes a pill?

21. What happens if I share my ART with a partner/friend/neighbor?

22. What if my partner refuses to use a condom?

23. My partner is also positive, are condoms still necessary?

24. What if I want to get pregnant while I am on ART?

Questions depicted in the tool were for illustrative purposes only. During the ‘Patient Empowerment’ training sessions, patients were encouraged to develop their own relevant questions.
