# Supplementary material for: “We Are Now Free to Speak”: Qualitative Evaluation of an Education and Empowerment Training for HIV Patients in Namibia
Source: PLoS One. 2016 Apr 7;11(4):e0153042. doi: 10.1371/journal.pone.0153042 (PMC4824517; doi:10.1371/journal.pone.0153042)
Supplement: S2 Fig — (DOCX) [file pone.0153042.s002.docx]

**Figure 2: Body Map Tool**

**Instructions**: Circle or shade in any areas on the body that have been affected by pain,

discomfort, rash, discoloring, odor or changes from normal.

**
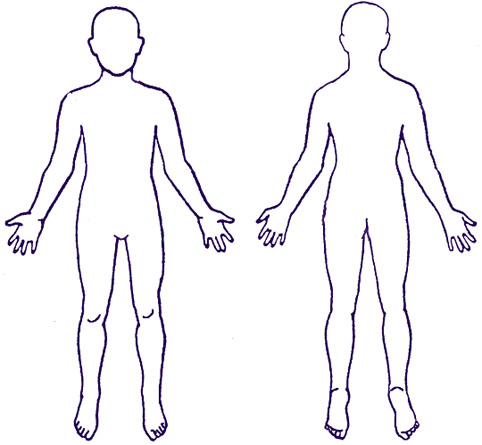
**
