# Supplementary material for: “We Are Now Free to Speak”: Qualitative Evaluation of an Education and Empowerment Training for HIV Patients in Namibia
Source: PLoS One. 2016 Apr 7;11(4):e0153042. doi: 10.1371/journal.pone.0153042 (PMC4824517; doi:10.1371/journal.pone.0153042)
Supplement: S3 Fig — (DOCX) [file pone.0153042.s003.docx]

**Figure 3: “I Tool”**

*
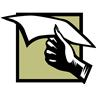
* **“I” Tool**

**Use these *example* statements (or others!) as a way to train yourself to be more assertive with your doctor or provider. Remember, your health is what is most important. You need to communicate!**

**Lifestyle Concerns**

1. I don’t have anyone to support me in taking ART.
2. I have not told my partner(s) about my HIV status.
3. I have not told my children about my HIV status.
4. I don’t understand why my husband is negative and I’m positive.
5. I’m really worried that I miss my pills too often.
6. I am too tired to work because of the pills and side effects.
7. I have other health problems that are making ART very difficult for me.

**Blood and Lab Work**

1. I don’t understand what CD4 count means.
2. I lost the paperwork that was given to me last time.
3. I don’t understand what is written in my ART card/passport.
4. I was not given any notice that these tests were going to be done today.
5. I wasn’t given the test results that I was promised.

**Medication and Condom Use**

1. I would like to know more about how these drugs work in my body.
2. I want to know more about which vitamin pills are safe to use.
3. I’m pregnant and I wasn’t pregnant last time. I need information about this.
4. I have too many side effects to continue taking these pills.
5. I want to switch to a new medicine. I can’t support the ones I have.
6. I don’t understand what the pharmacist told me.
7. I don’t understand what the community counselor told me.
8. I don’t know where to buy condoms.
9. I am having difficulty convincing my partner to use condoms.
10. I don’t understand why I need to take so many different kinds of pills.

**KEY ACTION PHRASES**

I can’t come back on that date.

I want to know if there is a treatment supporter or community counselor who can help me.

I need more time to talk to you.

I came a long way and waited a long time. I need more time to talk to you.

I don’t understand what you are saying.

I don’t understand your English well enough, can we call an interpreter?

I don’t like how the nurse treated me.
