# Supplementary figures and images for: “We Are Now Free to Speak”: Qualitative Evaluation of an Education and Empowerment Training for HIV Patients in Namibia
Source: PLoS One. 2016 Apr 7;11(4):e0153042. doi: 10.1371/journal.pone.0153042 (PMC4824517; doi:10.1371/journal.pone.0153042)

**Figure 4: Theory of Change Diagram**


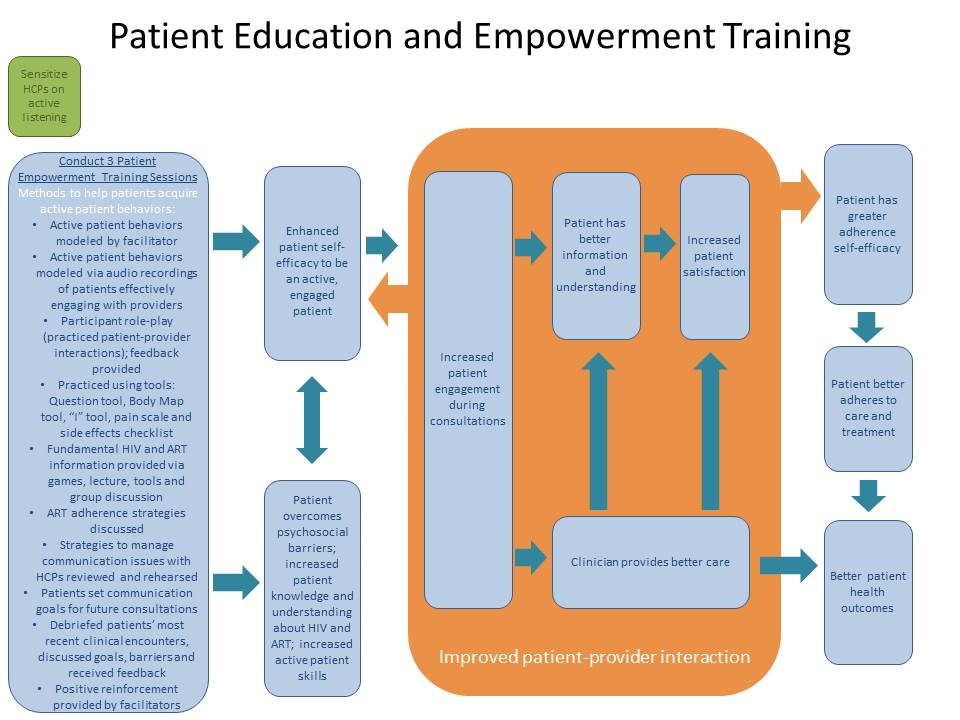

Supplement: S4 Fig — (DOCX) [file pone.0153042.s004.docx]
